# Fine wine, art, and medical physicist bibliometrics: Things that improve with age

**DOI:** 10.1002/acm2.70811

**Published:** 2026-09-25

**Authors:** Sundari Mehta, J John Lucido, Douglas Moseley, Catherine Holly Frank, Christopher L. Deufel

**Affiliations:** ^1^ Radiation Oncology Mayo Clinic Rochester Minnesota USA

**Keywords:** authorship inflation, bibliometrics, career age, medical physics, research productivity

## Abstract

**Purpose:**

Bibliometric indicators are widely used to evaluate faculty productivity, yet these metrics change with career stage, evolving authorship practices, and technological shifts in how science is produced. This study examines patterns of scholarly output among radiation therapy medical physics faculty at leading cancer centers and provides a framework and quantitative metrics for managing career age‐ and era‐based effects.

**Methods:**

Peer‐reviewed articles (1969–2025) were collected from Scopus for 397 Ph.D. medical physics faculty at the 20 U.S. cancer centers most highly ranked by US News and World Report in 2025. The bibliometric measures included annual rates of authorships ($\dot{A}$), first/last authorships ($\dot{FL}$), monograph‐equivalents ($\dot{ME}$), as well as the cumulative h‐index and contribution‐normalized ha‐index. Career age was defined by year of first publication. Productivity distributions were evaluated by career age using empirical cumulative distribution functions derived from 2016–2025 data. Era‐dependent fluctuations (1990–2025) were examined using age‐normalized metrics and trends in authorship team size. Tabulated bibliometrics as a function of career age are provided for evaluation of individual performance.

**Results:**

Productivity increased substantially with career age, peaking in late career. Senior faculty produced approximately two‐fold higher annual output than early‐career peers. At career ages of 1, 10, 20, 30, and 40 years, the median bibliometric rates were $\dot{A}$ = (1.2, 1.6, 2.5, 2.5,1.8) year^−1^, $\dot{FL}$ = (0.4, 0.4, 0.5, 0.5,0.2) year^−1^, $\dot{ME}$ = (0.2, 0.2, 0.3, 0.3,0.3) year^−1^, h‐index = (0, 7, 15, 23,43), and ha‐index = (0, 3.3, 6.0, 9.4,15.0). Era‐based effects were substantial. Between 1990 and 2025, median authors per paper increased from 4 to 10, inflating authorship‐based metrics. In contrast, contribution‐weighted measures (FL, ME, ha‐index) demonstrated greater temporal stability.

**Conclusions:**

Scholarly productivity in radiation therapy medical physics is strongly dependent on career age, with peak output occurring near the end of a typical career before declining modestly near retirement. Career‐age‐based percentile benchmarks provide a more balanced framework for evaluating academic performance than unadjusted bibliometric comparisons, especially for early‐career investigators. Due to changes in authorship norms over time, first/last authorships, monograph equivalents, and ha‐index may provide more accurate and equitable assessments of research performance than authorships or h‐index.

## 1 INTRODUCTION

Bibliometric indicators such as publication counts, authorship position, citation‐based indices, and related derivatives are widely used to assess academic productivity in medicine and the physical sciences.^1^ These metrics influence hiring, promotion, tenure decisions, institutional benchmarking, and allocation of research resources. Their appeal lies in their apparent objectivity, scalability, and ease of comparison across individuals and institutions. Bibliometrics serve as a convenient proxy for scholarly impact and engagement. However, overreliance on such metrics can obscure important dimensions of academic contribution.

Importantly, bibliometric measures can be imperfect representations of productivity when evaluated without regard to career stage, individual contributions, authorship norms, and historical context.^2^, ^3^ Publication practices evolve as fields mature, collaboration networks expand, and technological innovations alter how research is conducted and disseminated. A growing body of literature demonstrates that bibliometrics vary considerably with career age.^4^, ^5^ Early career investigators exhibit substantially lower publication rates than mid‐ and late‐career faculty as a result of experience and collaboration networks,^6^, ^7^ underscoring the importance of evaluating productivity relative to career age.^8^, ^9^ Bibliometrics are also strongly influenced by era‐dependent effects. Increases in authorship team size and citation density have inflated authorship metrics and h‐index over time.^8^, ^9^, ^10^ Recognizing and quantifying these age and era effects is essential for fair, meaningful interpretation of bibliometric performance.

Published studies of radiation oncology research productivity have primarily examined radiation oncologists. Zhang et al. reported wide variability in radiation oncologist bibliometric productivity and relationships between productivity, rank, department size, and funding.^10^ Choi et al. observed that h‐index strongly correlated with academic rank.^11^ Deufel et al. demonstrated strong age and era effects, including a substantial rise in bibliometrics over a career and an increase in the median number of authors per publication between 1990 and 2022.^8^ Gender and institutional differences among radiation oncologists have also been reported.^11^, ^12^, ^13^

Relatively little research exists on bibliometrics for radiation therapy medical physicists. Meeks et al. quantified bibliometric differences across degree type, academic rank, and gender to support promotion‐related decisions.^14^ Their analysis focused on cumulative measures of productivity and academic rank groupings, limiting insight into career‐age–specific trends.

This study extends the methods of Deufel et al. to address the limitations of prior bibliometric literature for academic radiation therapy medical physicists, specifically with regards to the concepts of age and era. Although the analytical framework parallels the methods previously developed for the analysis of radiation oncologists, the present study addresses a distinct academic discipline with different publication practices, collaboration patterns, and promotion expectations, requiring discipline‐specific benchmarks. The results quantify research productivity as a function of career age and include data tables that can be used to calculate the percentile ranking for a medical physicist according to their career age. Additionally, the study evaluates changes in bibliometrics of the field over time. The study focused on three primary objectives:

1. Provide charts to measure annual performance for academic radiation therapy medical physicists.
2. Develop tools to manage the confounder of career age with better granularity than academic rank.
3. Describe bibliometric differences across time in the radiation therapy medical physics field due to technological innovation and patterns of co‐authorship

## 2 METHODS AND MATERIALS

### 2.1 Institutions and faculty selection

**FIGURE 1:**
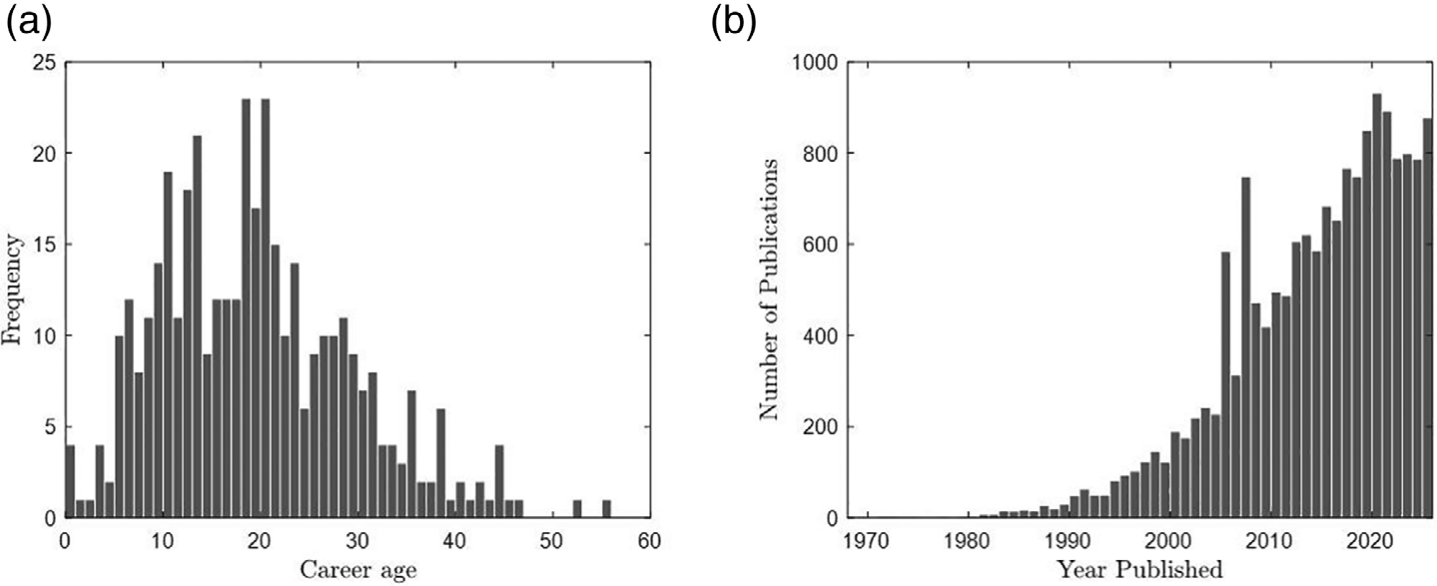
Histograms showing the distribution of career age and publication year for the study dataset. (a) Distribution of career ages for the 397 radiation therapy medical physics faculty included in the study. Career age was defined as the time elapsed between an individual's first publication and 2025. The distribution shows relatively few early career faculty (≤ 5 years) and late career faculty (≥ 40 years), reflecting the natural cycle of academic workforce entry and retirement. (b) Annual publication counts for the 16,319 publications authored by these faculty between 1969 and 2025.

A cohort of leading cancer institutions was compiled using the 2025 U.S. News & World Report Top 20 Cancer Center rankings.^15^ The institutions, in alphabetical order, were: Cedars‐Sinai Medical Center, City of Hope National Medical Center, Cleveland Clinic, Dana‐Farber Cancer Institute, Houston Methodist Hospital, The Johns Hopkins Hospital, The University of Texas MD Anderson Cancer Center, Massachusetts General Hospital, Mayo Clinic (Rochester, Minnesota), Memorial Sloan Kettering Cancer Center, Mount Sinai Hospital, New York‐Presbyterian/Columbia University Irving Medical Center and New York‐Presbyterian/Weill Cornell Medical Center, Northwestern Memorial Hospital, NYU Langone Hospitals, Stanford Hospital, UCLA Health (Ronald Reagan UCLA Medical Center), UCSF Health‐USCF Medical Center (University of California, San Francisco), University of Chicago Medicine, Penn Medicine (Hospital of the University of Pennsylvania), and The University of Texas Southwestern Medical Center. Within these institutions, 397 radiation oncology medical physics faculty were identified using departmental websites (Figure 1a). Inclusion was limited to faculty holding a PhD. Individuals based at satellite campuses or listed as emeritus were excluded to focus the analysis on actively practicing academic physicists.

### 2.2 Publication data

Publication data were obtained using Scopus, which is regarded as a leading bibliometric database with rigorous indexing standards, strong author‐disambiguation system, and broad coverage across biomedical, physical, and engineering sciences. Peer‐reviewed articles and reviews published between 1969 and 2025 were collected for all identified medical physics faculty members (Figure 1b). For each publication, the following information was collected: Authorship list and order, year of publication, cumulative citations as of 2025, and associated keywords. Publications with more than 50 authors were excluded to overcome the confounder of hyperauthorship. Medical physics faculty may have trained or worked in high‐energy physics, where the authorship culture of large experimental collaborations results in author lists that can exceed the thousands and where many coauthors have no direct involvement in the specific manuscript. This criterion ensured that authorship and citation patterns more accurately reflect meaningful contributions within medical physics.

### 2.3 Bibliometric analysis

The bibliometric approach used in this study follows the framework established by Deufel et al. for evaluating scholarly productivity as a function of career age and era.^8^ Several bibliometrics were selected to measure research volume and contribution. These included the annual rate of authorships ($\dot{A}$) with any position in the byline, the subset of publications for which a faculty member served as first or last author ($\dot{FL}$), and monograph equivalents ($\dot{ME}$). Monograph equivalent is a fractional credit system, where each publication (for which a faculty member held *any* position in the byline) was assigned a total value of one monograph equivalent, which was then divided among the authors according to their *position* in the byline. The weighting scheme followed the method originally described by Aziz and Rozing^16^ where contribution decreases progressively as author rank approaches the middle position.

(1) $$ME=\frac{1+\left|N+1-2R\right|}{\frac{1}{2}N^{2}+N\left(1-D\right)}\;$$

N equals the number of authors in the article, R is the rank of the author in the list (e.g., the first author is *R* = 1, the second author is *R* = 2, and the last author is *R* = N), and D equals 0 if N is an even number and $\frac{1}{2N}$ if N is an odd number. D is a parity correction factor that accounts for whether the number of authors is even or odd, ensuring the weighting function is properly normalized and symmetric.

Citation‐based metrics were also studied, including the h‐index and an ha‐index, which substitutes $\dot{ME}$ for authorship counts to yield a contribution‐normalized analogue of the h‐index.

(2) $$\begin{array}{cc} & \mathrm{ha}-\mathrm{index}\\ & \;=max\left\{count\left(\;ME*Citations\geq j\right)\geq j,\;j=0,\;0.1,\text{…}n\right\} \end{array}$$

To reduce year‐to‐year volatility and mitigate the arbitrariness of a January 1 cutoff date, all annual metrics were processed using a three‐year centered smoothing window. A ± 1‐year boxcar smoothing window was used when binning data by career age due to the limited number of faculty included in this study.

### 2.4 Career age and productivity

Career age–based metrics were computed by first defining the onset of an individual's research career (e.g., Career age = 0) as the calendar year in which their earliest publication appeared, irrespective of authorship position. Each subsequent year was assigned a career age equal to the number of years elapsed since that first publication.

(3) CareerAge=CurrentYear−YearofFirstPublication

For career ages 1 through 40, the mean and standard error of the mean were calculated for all bibliometrics, along with empirical cumulative distribution functions (ECDFs). The ECDF and mean analysis was limited to manuscripts published between 2016 and 2025, a period chosen to reflect contemporary patterns of scholarly activity.

**FIGURE 2:**
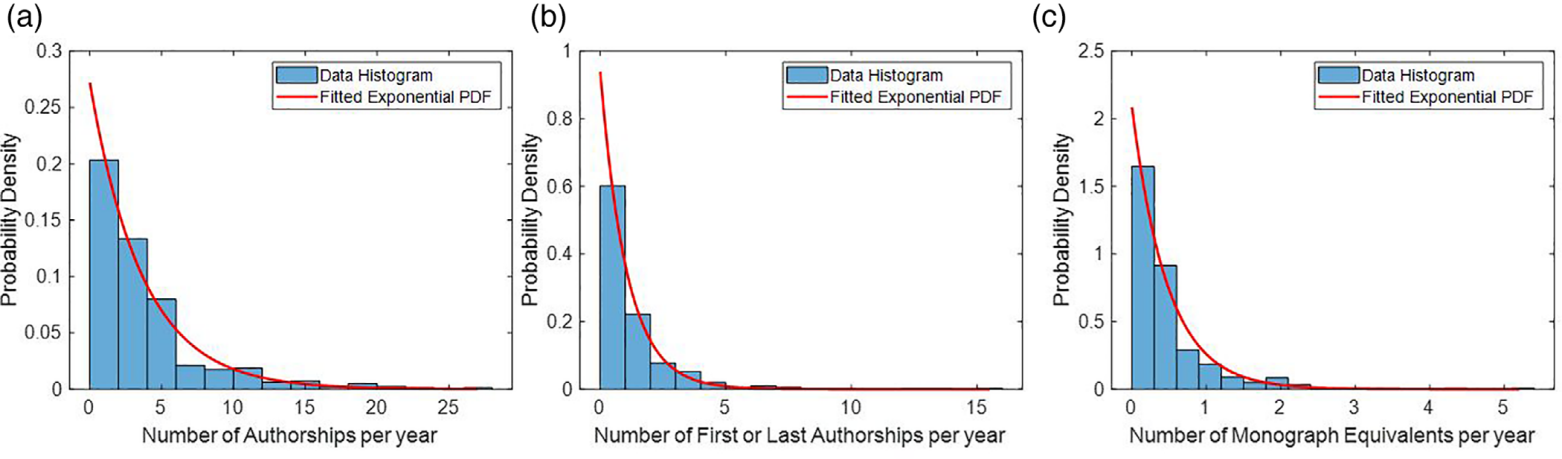
Example probability distributions showing bibliometric rates at career age equal to 20 years. Data is shown for (a)authorships per year, (b)first or last authorships per year, and (c)monograph equivalents per year. The red line in each plot represents a fit to the data using an exponential probability density function (PDF).

The distribution of $\dot{A}$, $\dot{FL}$, and $\dot{ME}$ within each career age was empirically consistent with an exponential probability density function (PDF), with an example shown in Figure 2 for the career age equal to 20 years. For a given bibliometric, M,

(4) $$PDF=\frac{1}{\mu \left(age\right)}\;*e^{-\frac{M}{\mu \left(\mathrm{age}\right)}\;}$$

(5) $$\mathrm{mean}\;=\mu \left(age\right)$$

The PDF is a useful tool for comparing faculty at different stages of their careers. Two researchers at different career ages, who fall within the same region of the PDF, can be viewed as performing at a comparable level, even if their raw publication counts differ substantially due to career age. Furthermore, a career age normalized bibliometric can be calculated using the age‐specific mean, μ(age).

(6) $$\begin{array}{cc} \hat{M} & =\frac{M}{\mu \left(age\right)},\;career\;age\;normalized\;metric\;when\;M\\ & \;follows\;an\;Exponential\;PDF \end{array}$$

In this context, a value of $\hat{M}$ equal to 1 indicates performance at the mean for one's career age, with higher or lower values representing above‐ or below‐average productivity, respectively.

### 2.5 Bibliometric fluctuations between 1990–2025

Bibliometric rates may change over time within a field due to differences in collaboration size, publication practices, and academic expectations that cause raw metrics to drift upward or downward. To account for these temporal shifts, referred to here as bibliometric fluctuation, we examined whether bibliometric productivity changed between 1990 and 2025.

$\dot{A}$, $\dot{FL}$, and $\dot{ME}$ bibliometrics were age‐normalized using Equation 6 with normalization constants obtained from the methods of Section 2.4. The purpose of the age‐normalization was to handle different distributions of career ages in each calendar year. For a particular year, the average age‐normalized bibliometric, $\bar{\;\hat{M}\;}\left(year\right)$, was calculated with Equation 7.

(7) $$\bar{\hat{M}}\left(year\right)=\frac{1}{N_{authors}\left(year\right)}\sum_{Authors(year)}\bar{\hat{M}}\left(year,\;age,author\right)$$

where age represents the career age of the author in that given year, $N_{authors}\left(year\right)$ is the total number of authors active in a given year, $\bar{\hat{M}}\;\left(year,\;age,author\right)=\frac{M(author,year)}{\mu (age,year)}$ calculated using Equation 6, and the summation was performed over the number of authors active during that given year.

We also analyzed trends in the median number of authors per publication, since expansion in authorship lists can elevate metrics such as total authorships or h‐index without increasing the number of distinct manuscripts produced.

### 2.6 Statistical methods

The error bars associated with mean $\dot{A}$, $\dot{Fl}$, and $\dot{ME}$ as a function of career age and variation over time were calculated from the standard error of the mean. Error bars for the median number of authors from 1990–2025 represented upper and lower quartiles associated with each date.

## 3 RESULTS

### 3.1 Author career ages and publication output

Across the 20 radiation oncology departments included in this study, 397 medical physics faculty produced a total of 16,319 publications between 1969 and 2025. The distribution of career ages and publication years (Figure 1) was notably uneven: relatively few faculty had career ages less than 5 or greater than 40, reflecting the lag between initial student publications and faculty appointments and natural patterns of retirement.

### 3.2 Research performance across career age

**FIGURE 3:**
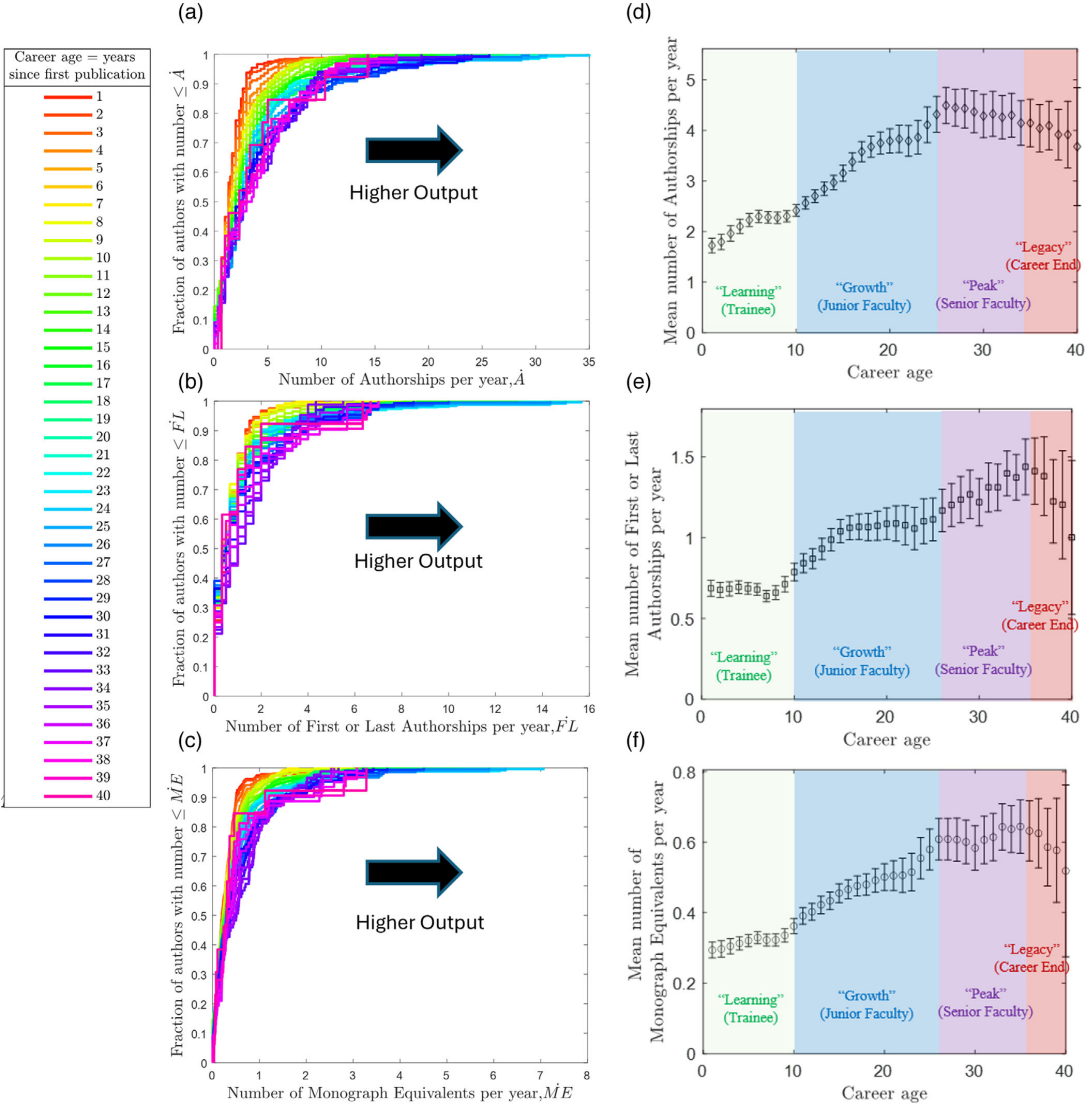
Empirical cumulative distribution functions for (a) authorships per year, (b) first or last authorships per year, and (c) monograph equivalents per year, derived from data between 2016–2025. These distributions enable assignment of age‐specific percentile ranks. The average value of each bibliometric as a function of a career age is provided in figures (d–f), where shading is used to categorize how scholarly productivity in medical physics progresses through four broad phases, an early learning phase (green) with gradual output, a midcareer growth phase (blue) marked by steady increases, a late career peak (purple) characterized by sustained high productivity, and a final legacy phase (red) in which contributions decline modestly as retirement approaches. Error bars represent the standard errors of the mean.

**FIGURE 4:**
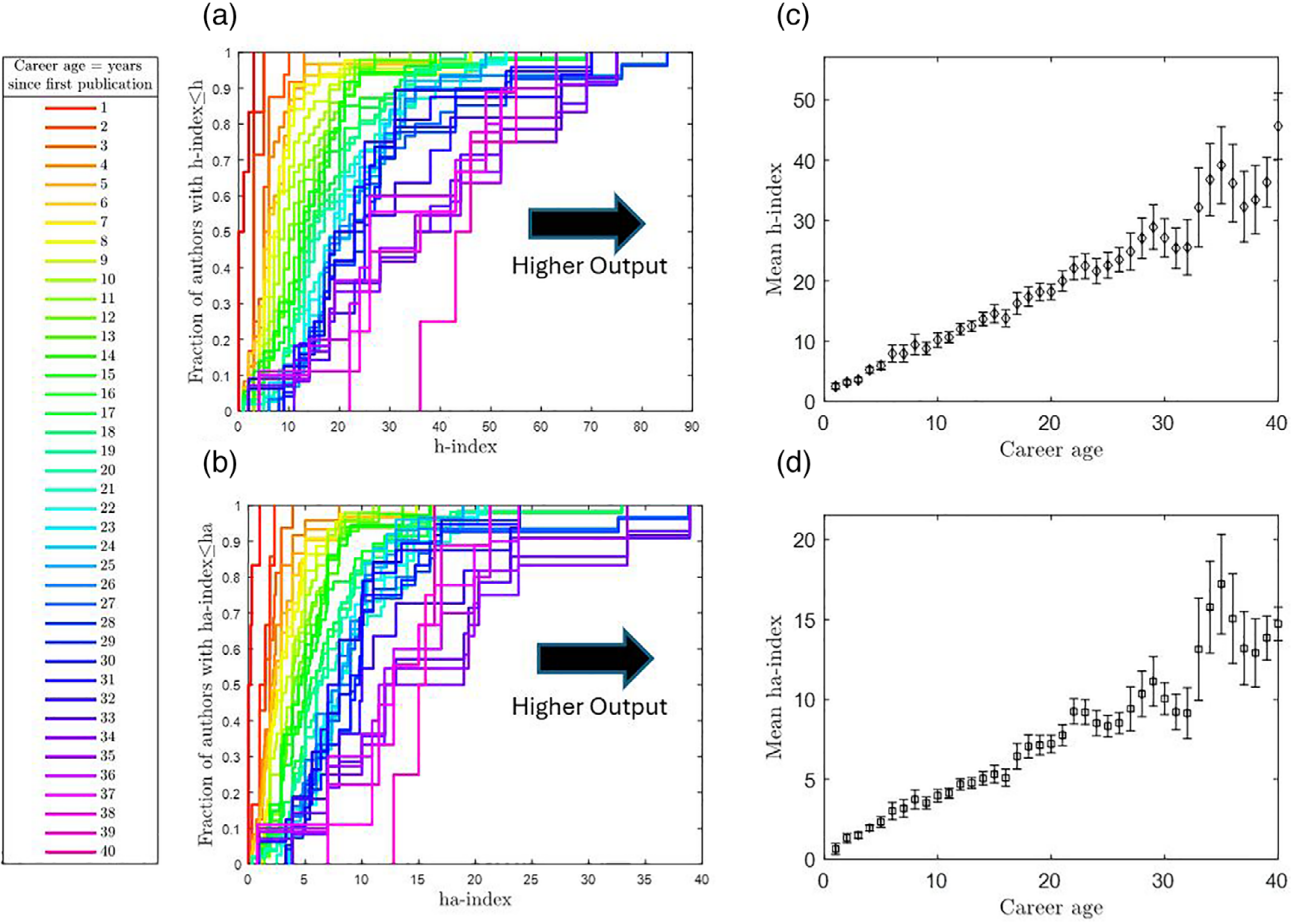
h‐index and ha‐index distribution as a function of career age. Empirical cumulative distribution functions for (a) h‐index and (b) ha‐index according to career age. The average value of each bibliometric as a function of a career age is provided in figures (c–d).

Age‐stratified bibliometric distributions are provided in Figures 3 and 4, and their CDF percentile values are reported in Table 1. Together, these cumulative distribution functions provide a framework for assigning percentile ranks to individual faculty members. Figures 3 a,b,c provide CDFs for $\dot{A}$, $\dot{FL},$ and $\dot{ME}$ that describe how the magnitude and distribution (e.g., variation in performance among faculty with the same career age) of bibliometric productivity evolves with career age. Figures 3 d,e,f presents the average values for $\dot{A}$, $\dot{FL},$ and $\dot{ME}$ at each career age. The analogous results for h and ha‐index are provided in Figure 4.

**TABLE 1:** Faculty productivity tables with percentile rank as a function of career age for the annual rates of authorships, first/last authorships, monograph equivalents, h‐index, and ha‐index.

	Career Age = Years since initial publication								
Percentiles	1	5	10	15	20	25	30	35	40
Any a uthorships									
**99**	10.4	12.5	10.8	13.9	23.3	29.2	23.8	16.4	13.8
**95**	3.4	6.5	7.3	10.8	11.8	13.6	14.4	12.0	11.7
**90**	2.8	4.9	5.5	7.0	8.5	9.9	9.3	9.3	8.7
**80**	2.2	3.0	3.6	4.7	5.3	6.0	7.0	6.8	4.7
**70**	1.8	2.5	2.8	3.7	3.9	3.9	4.7	5.5	3.5
**60**	1.5	1.8	2.2	2.7	3.0	3.0	3.3	3.8	2.9
**50**	1.2	1.5	1.6	2.0	2.5	2.6	2.5	2.7	1.8
**40**	0.9	1.0	1.1	1.5	1.9	2.1	1.7	1.7	1.1
**30**	0.7	0.7	0.7	0.9	1.3	1.6	1.3	1.0	0.7
**20**	0.5	0.4	0.4	0.6	0.7	0.8	0.8	0.6	0.7
**10**	0.4	0.0	0.0	0.0	0.1	0.3	0.2	0.1	0.7

**Average**	1.7	2.2	2.4	3.2	3.8	4.3	4.3	4.1	3.7
**First or l ast a uthorships**									
**99**	4.1	3.2	5.7	6.9	9.1	9.2	6.8	5.8	5.8
**95**	1.7	2.0	2.7	4.0	4.4	4.2	5.4	4.1	3.5
**90**	1.3	1.6	1.7	2.8	2.5	2.9	3.6	3.6	1.8
**80**	1.0	1.0	1.0	1.5	1.4	1.5	1.8	2.3	1.1
**70**	0.7	0.7	0.7	0.9	0.9	0.9	1.1	1.7	0.7
**60**	0.6	0.6	0.6	0.7	0.6	0.6	0.7	1.2	0.3
**50**	0.4	0.4	0.4	0.5	0.5	0.4	0.5	0.8	0.2
**40**	0.3	0.2	0.1	0.2	0.2	0.1	0.1	0.6	0.1
**30**	0.1	0.0	0.0	0.0	0.0	0.0	0.0	0.2	0.0
**20**	0.0	0.0	0.0	0.0	0.0	0.0	0.0	0.0	0.0
**10**	0.0	0.0	0.0	0.0	0.0	0.0	0.0	0.0	0.0

**Average**	0.7	0.7	0.8	1.0	1.1	1.1	1.2	1.4	1.0
**Monograph equivalents**									
**99**	2.0	1.5	2.4	2.8	3.0	4.2	3.2	2.5	3.0
**95**	0.7	1.1	1.2	1.5	1.9	2.0	2.2	2.3	1.9
**90**	0.5	0.7	0.7	1.0	1.0	1.4	1.4	1.6	0.9
**80**	0.4	0.5	0.5	0.7	0.7	0.8	0.8	1.0	0.4
**70**	0.4	0.3	0.4	0.5	0.5	0.5	0.5	0.8	0.3
**60**	0.3	0.3	0.3	0.4	0.4	0.4	0.4	0.6	0.3
**50**	0.2	0.2	0.2	0.3	0.3	0.3	0.3	0.5	0.3
**40**	0.2	0.2	0.2	0.2	0.2	0.2	0.2	0.3	0.1
**30**	0.1	0.1	0.1	0.1	0.2	0.2	0.2	0.2	0.1
**20**	0.1	0.1	0.1	0.1	0.1	0.1	0.1	0.1	0.1
**10**	0.0	0.0	0.0	0.0	0.0	0.0	0.0	0.0	0.0

**Average**	0.3	0.3	0.4	0.5	0.5	0.6	0.6	0.6	0.5
**h‐index**									
**99**	3	13	41	39	42	49	66	74	55
**95**	3	11	21	29	36	44	54	71	53
**90**	2	10	17	22	33	33	49	68	51
**80**	2	8	13	19	24	29	32	59	48
**70**	1	6	10	17	21	26	28	47	45
**60**	1	5	8	16	19	23	26	42	44
**50**	0	5	7	14	15	21	23	35	43
**40**	0	5	7	11	13	17	19	26	40
**30**	0	4	6	8	12	16	16	19	37
**20**	0	3	5	7	10	14	14	16	36
**10**	0	1	3	4	8	9	11	12	36

**Average**	3	6	10	15	18	23	27	39	46
**ha‐index**									
**99**	1.0	7.3	13.1	15.2	19.2	17.4	22.2	38.2	16.4
**95**	0.8	4.8	7.6	10.4	14.1	12.8	16.9	35.6	16.2
**90**	0.6	3.7	6.9	8.0	13.4	11.6	16.2	31.5	16.1
**80**	0.3	2.8	5.0	6.9	9.2	10.2	13.7	22.4	15.8
**70**	0.2	2.5	4.7	6.3	8.0	9.5	10.1	19.8	15.5
**60**	0.1	2.3	3.8	5.4	7.0	8.8	9.9	19.1	15.2
**50**	0.0	1.9	3.3	4.9	6.0	7.8	9.4	12.0	15.0
**40**	0.0	1.7	2.7	4.2	5.5	7.3	8.3	10.8	14.1
**30**	0.0	1.2	2.0	2.9	4.9	6.1	6.9	8.8	13.2
**20**	0.0	1.0	1.7	2.8	3.9	4.8	5.6	6.1	12.8
**10**	0.0	0.5	1.4	1.8	3.1	3.9	4.3	4.2	12.8
**Average**	**0.7**	**2.3**	**4.0**	**5.3**	**7.2**	**8.3**	**10.1**	**17.2**	**14.7**

Authorship‐based productivity was observed to rise rapidly during early career stages and peak near the end of a typical career. The average bibliometric productivity at the most productive career age increased by 161% for $\dot{A}$ (Figure 3d), 110% for $\dot{FL}$ (Figure 3e), and 119% for $\dot{ME}$ (Figure 3f) compared to career age 1. Average $\dot{A}$ increased from 1.7 per year at career age 1 to a peak of 4.5 per year at career age 26 and remained greater than 4 through career age 37 (Figure 3d). Average first/last authorships followed a slower trajectory, starting at 0.7 per year, peaking at 1.44 per year at career age 35, and then declining to 1 by career age 40 (Figure 3e). Average monograph equivalent (ME) contributions began at 0.29 per year, increased steadily to a maximum of 0.64 per year at career age 35, and tapered to 0.52 by career age 40 (Figure 3f). Both h‐index and ha‐index increased approximately linearly with career age, with the h‐index increasing at 0.93 units per year (*R* ^2^ = 0.96) and the h_a_‐index rising at 0.36 units per year (*R* ^2^ = 0.93) (Figure 4 c, d).

**FIGURE 5:**
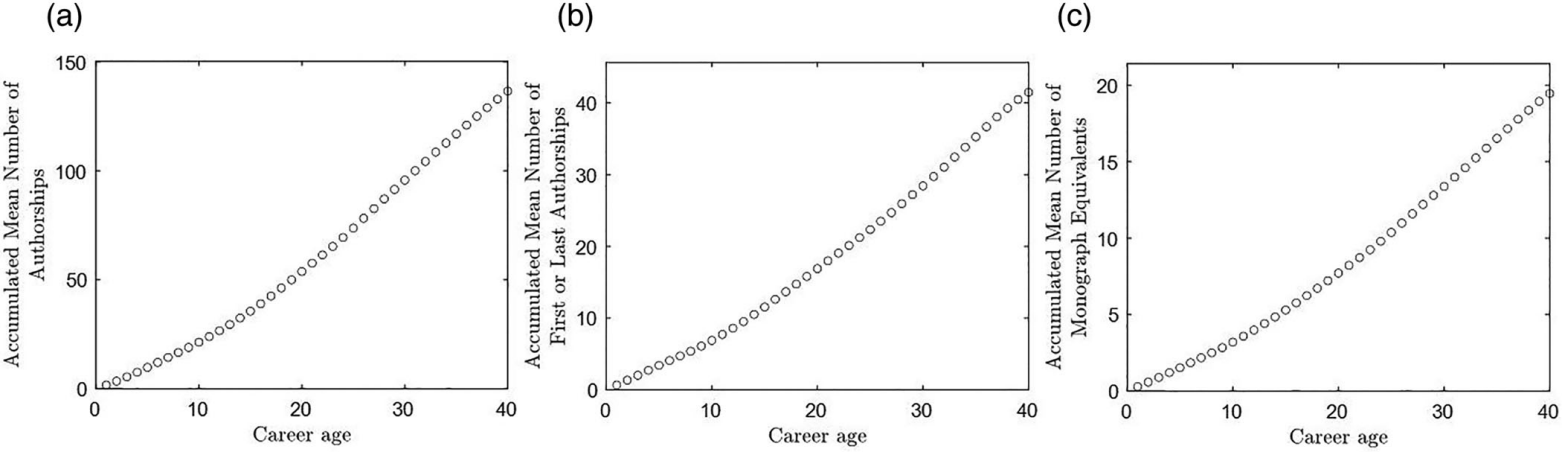
Average accumulated bibliometrics for (a) authorships (b) first/last authorships, and (c) monograph equivalents as a function of career age. The regions with convex curvature signify productivity rates that increase with career age. The data was obtained by integrating the average bibliometric rates from Figure 3.

As illustrated in Figure 3, these patterns reflect four approximate phases of academic development: an early learning phase spanning career ages 0–10, during which $\dot{ME}$ and $\dot{FL}$ remain stable while $\dot{A}$ slowly increases as individuals begin conducting research and engage in collaborative work; a growth phase during mid‐career in career ages 11–25 when $\dot{A}$, $\dot{FL},$ and $\dot{ME}$ increase nearly monotonically; a peak phase for senior faculty in career ages 26–35, marked by a plateau in $\dot{A}$ and $\dot{ME}$, and continued gains in $\dot{FL}$; and a late‐career legacy phase beginning after career age 35, characterized by modest declines in $\dot{A}$ and $\dot{ME}$, and sharper reductions in $\dot{FL}$ contributions. Figure 5 summarizes the average accumulated bibliometrics for $\dot{A}$, $\dot{FL},$ and $\dot{ME}$ as a function of career age, obtained by integrating the average bibliometric rates from Figure 3. This is a useful representation of the data for measuring how cumulative performance compares against the average. The regions with convex curvature in Figure 5 signify that average productivity rates increase with career age.

### 3.3 Percentile use case

Table 1 may be used to evaluate an individual's performance in a particular year relative to peers of the same career age. For example, consider a faculty physicist with career age equal to 15 who in 2025 had 2 authorships, 1 first or last authorships, 0.5 $\dot{ME}$, an h‐index of 15, and ha‐index of 5. According to Table 1, their performance would rank at the 50^th^ percentile for authorships, the 72^nd^ percentile for first or last authorships, the 70^th^ percentile for $\dot{ME}$, the 55^th^ percentile for h‐index, and the 52^nd^ percentile for ha‐index.

### 3.4 Field‐wide patterns over time

**FIGURE 6:**
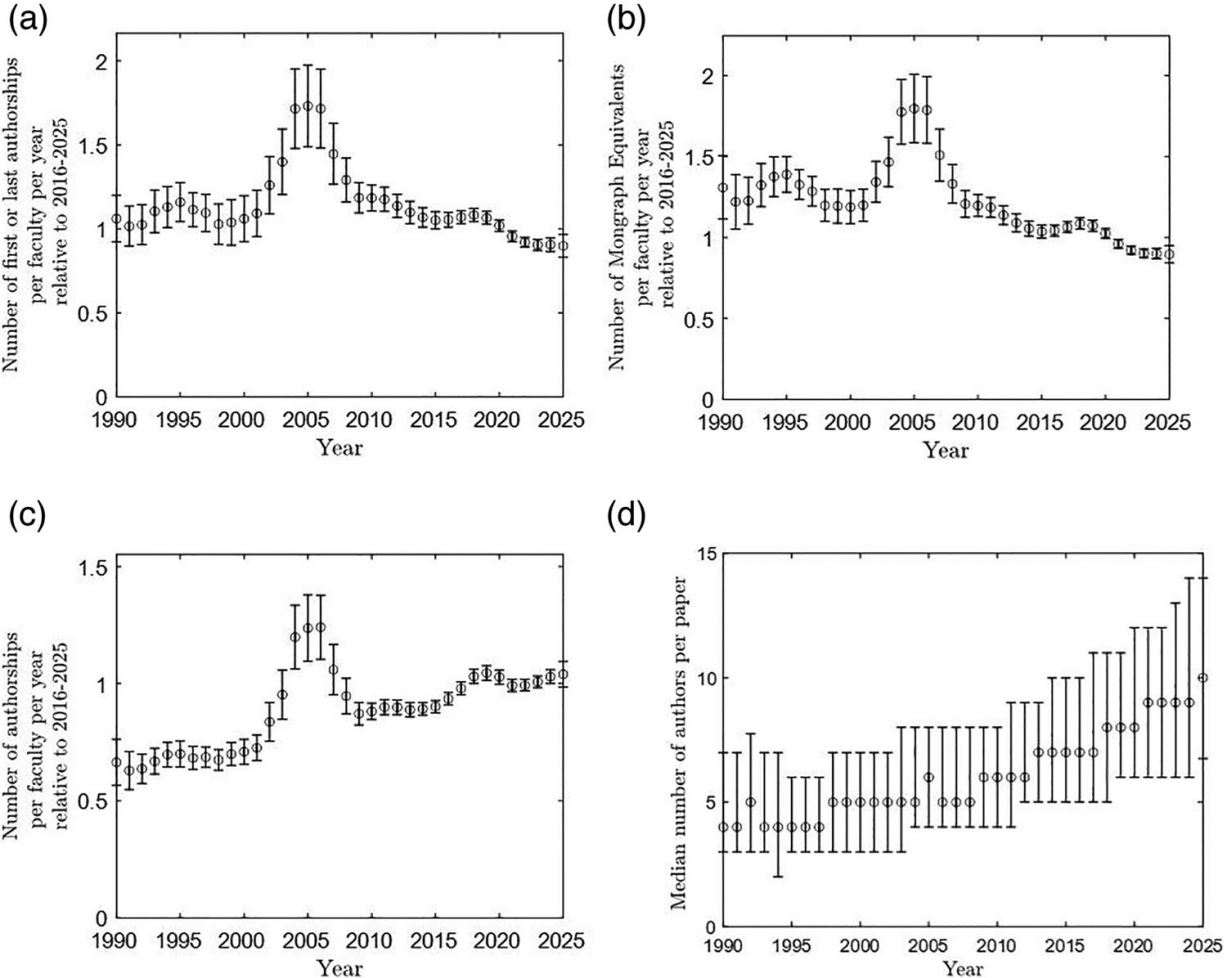
Radiation therapy medical physics bibliometric trends 1990–2025. Trends in rates of (a) first/last‐authorships, (b) monograph‐equivalents, and (c) authorships. Error bars represent the standard errors of the mean. (d) The median number of authors per publication indicates bibliometric inflation over the study period. Error bars represent upper and lower quartiles associated with each date.

An analysis of bibliometric trends between 1990 and 2025 revealed changes in authorship‐based productivity metrics across the 35‐year time window. Figure 6 illustrates that rates of $\dot{FL}$ (Figure 6a) and $\dot{ME}$ (Figure 6b) were comparable in 1990 and 2025, with several notable peaks in the intervening years, possibly linked to major technological developments such as electronic treatment planning, IMRT, IGRT, and the emergence of artificial intelligence. One might speculate on future innovation “waves” that result from new technology or changes in data collection or manuscript preparation efficiency (e.g., artificial intelligence).

For example, a pronounced productivity surge was observed between 2003–2007, during which $\dot{FL}$ authorships were 73% higher than the period 2016–2025 (Figure 6a). An analysis of the manuscript keywords from this time period revealed an increase in the appearance of publications with keywords related to computed tomography, motion management, and IMRT compared with earlier periods. Authorships (Figure 6c) displayed a similar oscillatory pattern with a peak that was 24% higher and superimposed on a gradually rising baseline that increased by approximately 1% per year. This rising baseline was attributed to the growth in authorship list lengths, where between 1990 and 2025, the median number of authors per publication increased from 4 to 10 (Figure 6d).

## 4 DISCUSSION

This study provides the first career‐age–resolved and era‐adjusted analysis of scholarly productivity for radiation therapy medical physicists across top U.S. cancer centers, offering a view of how research output evolves over the academic lifespan and how publication norms have shifted over time. By quantifying authorship counts, first/last‐author contributions, $\dot{ME}$, and citation‐based indices for 397 faculty members, benchmarks were established for the evaluation of bibliometric productivity. These benchmarks highlight how productivity increases from early‐ to late‐career, with peak output occurring near retirement, thereby underscoring the continued scholarly value of experienced physicists.

Cross disciplinary comparisons with radiation oncologists^8^ (e.g. comparison with faculty who have a different role within the same subspecialty) and dermatologists^9^ (e.g. comparison with faculty who have a different role in a different subspecialty) further demonstrate that productivity expectations vary markedly. For radiation therapy medical physicists, at career ages of 1, 10, 20, and 30, years, the median bibliometric rates were $\dot{A}$ = (1.2, 1.6, 2.5, 2.5) year^−1^, $\dot{FL}$ = (0.4, 0.4, 0.5, 0.5) year^−1^, $\dot{ME}$ = (0.2, 0.2, 0.3, 0.3) year^−1^, h‐index = (0, 7, 15, 23), and ha‐index = (0, 3.3, 6.0, 9.4).  The published metrics for the same career ages for academic dermatologists were reported to be greater, and with some metric and age combinations that were 120% greater: $\dot{A}$= (1.0, 1.7, 3.4, 3.4) year^−1^,  $\dot{FL}$= (0.4, 0.6, 1.1, 0.6) year^−1^,  $\dot{ME}$= (0.3, 0.4, 0.6, 0.4) year^−1^, h‐index = (0, 6, 16, 26), and ha‐index = (0.5, 3.4, 7.9, 12.6). A similar pattern was observed for radiation oncologists, who had even greater raw metrics: $\dot{A}$= [1.5, 4.1, 6.5, 7.0] year^−1^, $\dot{FL}$= [0.5, 0.9, 1.2, 0.6] year^−1^, $\dot{ME}$= [0.2, 0.5, 0.7, 0.8] year^−1^,h‐index = [1, 12, 22, 47], and ha‐index = [0.4, 4.4, 6.9, 18.4]. As such, benchmarks drawn from one discipline may be inappropriate or misleading when applied to another. Some fields may require greater effort to generate data and publish, and expectations for scholarly output should differ correspondingly. These results reinforce the importance of using field specific metrics and interpreting performance with respect to career age.^17^

The increase in authorships and median number of authors per paper between 1990 and 2025 was consistent with bibliometric inflation reported among radiation oncologists and reinforced the challenges with cross‐era comparisons.^18^ Promotion and tenure committees are advised to carefully consider bibliometric inflation in authorship metrics. Many senior faculty established their careers when small author teams were commonplace and a different cutoff for academic promotion should be considered for a metric using authorships and h‐index. Contribution‐focused measures such as first/last authorships, $\dot{ME}$, and ha‐index appeared to be more stable and may better ensure modern faculty are evaluated fairly against historical standards.

Ongoing monitoring of bibliometric trends is necessary to ensure that expectations remain relevant and equitable. In this context, Goodhart's law (“When a measure becomes a target, it ceases to be a good measure“) and CampbNoneell's law (”The more a quantitative metric indicator is used for decision making, the more it corrupts the process it is intended to monitor”) highlight the risks of quantitative metrics for academic evaluation.^19^ Metrics should be applied to motivate meaningful scholarly contribution rather than incentivizing strategic behavior. Age and era‐aware interpretations, combined with contribution‐based metrics, provide a more reliable framework for assessing academic performance.

A choice was made to exclude papers with 50 or more coauthors to eliminate the confounder of hyperauthorship. The cutoff value sensitivity was tested, and we observed no change using a cutoff of 100 authors, and overall, the cutoff had minimal impact on average metrics (e.g., a maximum decrease of less than 0.3 authorships per year across all ages and percentiles). Nevertheless, the cutoff did have a large effect on the metrics of a small number of authors. For example, the cutoff decreased the h‐index for one physicist from 39 to 18 and another physicist decreased from 30 to 13. This further underscored the usefulness of contribution‐based bibliometrics such as ha‐index. The authorship cutoff produced no change in the first author's ha‐index, and the second author's ha‐index decreased minimally, from 4.3 to 4.1.

The choice of smoothing window size did not affect the broad patterns (e.g., age‐based curve shape, peak region, magnitudes), compared without smoothing, but did reduce variation among neighboring elements and the size of error bars by increasing the size of each career‐age bin.

This study included institutions with a wide range of faculty sizes (e.g., smallest equal to 6 and largest equal to 67). We considered whether larger departments may have greater bibliometric rates due to increased collaboration opportunities. No statistically significant correlation (e.g., *p* < 0.05) was observed between faculty size and age‐adjusted percentile performance for $\dot{A}$, $\dot{FL},$ or $\dot{ME}$. This suggests that the intra‐institution variation among faculty was much greater than the inter‐institution variation. One possible explanation for this finding is that the clinical workload of medical physics teams is largely fixed and cannot be readily adjusted, thereby limiting the resources available for research.

This study has several limitations. First, the bibliometric dataset represents a limited cohort from highly ranked academic cancer centers that may not represent the broader medical physics community, particularly with regards to resources and protected research time compared with smaller or non‐academic centers. As such, the productivity levels reported here are best interpreted as benchmarks for faculty working in medium‐to‐large clinics with an academic focus. Second, career age was defined using the date of first publication. This proxy is imperfect, however more accurate indicators such as date of residency completion or board certification were not publicly accessible. First publication remains a reasonable approximation of research career onset and is likely well correlated with key career milestones. No distinction was made between publications in medical physics versus other physics subfields due to the lack of a reliable filter for this separation. Because publication practices and productivity expectations vary across disciplines, this limitation may have influenced estimates of early‐career productivity. This study also lacked subgroup analyses based on proportion of clinical versus administrative duties, research appointment, or gender, as such information was not publicly available.

## 5 CONCLUSIONS

This study establishes bibliometric benchmarks for radiation therapy medical physicists and demonstrates that research productivity improves with career age, peaking late in the academic career. Era dependent changes, including larger authorship teams, were shown to inflate traditional metrics such as total authorships and the h‐index, underscoring the limitations of cumulative or citation‐based measures for cross‐era comparisons. Contribution focused metrics, such as first/last authorships and $\dot{ME}$, provided more stable alternatives. Together, these findings provide a practical framework for interpreting bibliometric performance that accounts for both career stage and changing publication practices, thereby supporting more equitable academic evaluation.

## AUTHOR CONTRIBUTIONS

**Sundari Mehta**: Investigation; data curation; writing—original draft; writing—review and editing. **J. John Lucido III**: Conceptualization; methodology; writing—review and editing. **Douglas Moseley**: Conceptualization; methodology; writing—review and editing. **Catherine Holly Frank**: Methodology; investigation; data curation; formal analysis; software; visualization; writing—review and editing. **Christopher L. Deufel**: Conceptualization; methodology; investigation; data curation; formal analysis; software; validation; visualization; writing—original draft; writing—review and editing; supervision; project administration.

## Funding information

No funding was received for the work performed in this investigation.

## CONFLICT OF INTEREST STATEMENT

The authors declare no conflicts of interest.

## ETHICS STATEMENT

Ethical review and approval were not required for this study because it analyzed publicly available publication and citation data and involved no human subjects or identifiable patient information.

## DECLARATION OF GENERATIVE AI AND AI‐ASSISTED TECHNOLOGIES IN THE MANUSCRIPT PREPARATION PROCESS

All content and analysis were produced by the authors. During the preparation of this work the authors used ChatGTP version 5.3 for grammar and minor language editing assistance. After using this tool, the authors reviewed and edited the content as needed and take full responsibility for the content of the published article.

## Data Availability

Research data are stored in an institutional repository and will be shared upon request by the corresponding author.
